# Whole genome sequence of two *Rathayibacter toxicus* strains reveals a tunicamycin biosynthetic cluster similar to *Streptomyces chartreusis*

**DOI:** 10.1371/journal.pone.0183005

**Published:** 2017-08-10

**Authors:** Aaron J. Sechler, Matthew A. Tancos, David J. Schneider, Jonas G. King, Christine M. Fennessey, Brenda K. Schroeder, Timothy D. Murray, Douglas G. Luster, William L. Schneider, Elizabeth E. Rogers

**Affiliations:** 1 Foreign Disease/Weed Science Research Unit, Agricultural Research Service, U.S. Dept. of Agriculture, Frederick, Maryland, United States of America; 2 Emerging Pests and Pathogens Research Unit, Agricultural Research Service, U.S. Dept. of Agriculture, Ithaca, New York, United States of America; 3 Dept. of Entomology, Plant Pathology and Nematology, University of Idaho, Moscow, Idaho, United States of America; 4 Dept. of Plant Pathology, Washington State University, Pullman, Washington, United States of America; Academia Sinica, TAIWAN

## Abstract

*Rathayibacter toxicus* is a forage grass associated Gram-positive bacterium of major concern to food safety and agriculture. This species is listed by USDA-APHIS as a plant pathogen select agent because it produces a tunicamycin-like toxin that is lethal to livestock and may be vectored by nematode species native to the U.S. The complete genomes of two strains of *R*. *toxicus*, including the type strain FH-79, were sequenced and analyzed in comparison with all available, complete *R*. *toxicus* genomes. Genome sizes ranged from 2,343,780 to 2,394,755 nucleotides, with 2079 to 2137 predicted open reading frames; all four strains showed remarkable synteny over nearly the entire genome, with only a small transposed region. A cluster of genes with similarity to the tunicamycin biosynthetic cluster from *Streptomyces chartreusis* was identified. The tunicamycin gene cluster (TGC) in *R*. *toxicus* contained 14 genes in two transcriptional units, with all of the functional elements for tunicamycin biosynthesis present. The TGC had a significantly lower GC content (52%) than the rest of the genome (61.5%), suggesting that the TGC may have originated from a horizontal transfer event. Further analysis indicated numerous remnants of other potential horizontal transfer events are present in the genome. In addition to the TGC, genes potentially associated with carotenoid and exopolysaccharide production, bacteriocins and secondary metabolites were identified. A CRISPR array is evident. There were relatively few plant-associated cell-wall hydrolyzing enzymes, but there were numerous secreted serine proteases that share sequence homology to the pathogenicity-associated protein Pat-1 of *Clavibacter michiganensis*. Overall, the genome provides clear insight into the possible mechanisms for toxin production in *R*. *toxicus*, providing a basis for future genetic approaches.

## Introduction

There are few phytobacteria with the capacity to directly affect the health of humans or livestock. In the rare instances where they can, the pathogenic effects are often related to the production of toxins. One such toxin-producer is the Gram-positive bacterium *Rathayibacter toxicus*, the causative agent of annual rye grass toxicity (ARGT) in Australia. ARGT is an often-fatal toxicosis of forage animals caused by ingestion of infected hay or grain. Over 10 million hectares of Western Australian farmland has been affected and ARGT caused an estimated $40 million AUD in direct losses in 2010 [1]. *R*. *toxicus* produces a highly lethal tunicaminyluracil class corynetoxin (LD_50_ 3–5 mg/kg in sheep) that causes severe and often fatal neurological and hepatic disease [2]. Sub-lethal doses are also damaging to livestock and diminish wool quality and quantity, meat quality, and cause fetal abortions in sheep [1]. Symptom onset can occur up to 12 weeks after ingestion and a single exposure can cause lethality; toxin effects are cumulative [2]. *R*. *toxicus* corynetoxins were identified as a new member of the tunicaminyluracil class of antibiotics, which inhibit an early stage in prokaryotic peptidoglycan cell wall assembly [3]. In eukaryotes, tunicamycin reduces protein N-glycosylation by inhibiting uridine diphospho-*N*-acetylglucoseamine:dolichol-*N*-acetylglucoseamine-1-phosphate transferase [4]. The dangers to U.S. agriculture presented by *R*. *toxicus* and tunicamycin production in forage resulted in the bacterium being listed as a U.S. Department of Agriculture (USDA) Plant Protection and Quarantine Select Agent in 2008 and relisted in 2012 (www.selectagents.gov/SelectAgentsandToxinsList.html).

*R*. *toxicus* is most commonly found in annual ryegrass (*Lolium rigidum*) in association with *Anguina funesta* or other anguinid seed-gall nematodes. The infection cycle begins with *R*. *toxicus* adhering to the cuticle of compatible juvenile nematodes in the soil and being carried to the growing point of the forage grass. Once in a developing seed, the nematode and bacteria compete to form either a nematode or a bacterial gall. *R*. *toxicus* growth in developing galls can produce a yellow exopolysaccharide “slime” or gummosis; therefore, the plant infection is commonly called yellow slime disease. The trigger for toxin production is unknown but toxin generally appears late in the growing season as seed are senescing. Senesced seed, nematode galls, and bacterial galls dry and fall to the ground to repeat the disease cycle the following year. Host range of *R*. *toxicus* appears to be determined by the host range of the vectoring nematode [5, 6]. Tunicamycin production is often associated with the presence of an *R*. *toxicus*-specific bacteriophage NCPPB 3778, although toxin production has also been measured in the absence of phage [7, 8]. The NCPPB 3778 genome has recently been sequenced and is similar to siphoviral genomes [9]. Although its role in nature is unclear, NCPB3778 infection of *R*. *toxicus* can restore tunicamycin production in the lab, where the ability to produce tunicamycin is otherwise rapidly lost in culture (A.J. Sechler, personal observation).

Although complete genome sequences are publically available for two *R*. *toxicus* strains, FH-145 (NZ_CP010848.1) and WAC3373 (NZ_CP013292.1) [10], neither sequence has been carefully annotated. In addition, the full genetic diversity of *R*. *toxicus* is not well represented by these two strains alone [10, 11]. Therefore, two additional strains of *R*. *toxicus*, FH-79 (the type strain) and FH-232 were sequenced. Because an established system for genetic modification of *R*. *toxicus* is not available, the analysis presented here uses comparative and structural genomics to identify the genetic basis of several previously described phenotypes including the production of tunicamycin.

## Materials and methods

### Bacterial strains, culture, and DNA extraction

Cultures of *R*. *toxicus* FH-79 and FH-232 were obtained from Dr. Ian Riley (University of Adelaide, South Australia); additional information about their origins is presented in Table 1. *R*. *toxicus* was maintained on modified YGM (mYGM) [12]. One liter of this modified media contained yeast extract 2 g, glucose 1.25 g, K_2_HPO_4_ 0.25 g, KH_2_PO_4_ 0.25 g, MgSO_4_·7H_2_O 0.1 g, and agar 16 g. Cultures were incubated at 25°C unless otherwise noted; cryogenic stocks were stored in 15% glycerol at -80°C. DNA was extracted using a modified Marmur method [13] from 3 day old liquid cultures. DNA quality was estimated by OD_260/280_ ratio as measured on a Nanodrop 2000 (Thermo Fisher Scientific) and only DNA with a ratio >1.6 was used for sequencing. Purity of the cultures used for DNA extraction was confirmed by plating 50 μl on mYGM and monitoring for growth of non-*R*. *toxicus* colonies. 16S rDNA was sequenced using an Applied Biosystems 3130XL (Thermo Fisher Scientific) to test purity of extracted DNA prior to genomic sequencing; only extracted DNA yielding a single 16S sequence was sequenced further.

**Table 1:** Genome comparisons of sequenced *Rathayibacter toxicus* strains.

Strains	*R*. *toxicus* FH-79	*R*. *toxicus* FH-232	*R*. *toxicus* FH-145	*R*. *toxicus* WAC3373
alternate names	FH-137; CS14; ATCC49908	FH-100; CS37; SE3	70137; CS30	WSM194
reference	this study	this study	GenBank CP010848	[10]
chromosome size (bases)	2,343,780	2,394,755	2,328,288	2,346,032
GC content (%)	61.5	61.4	61.5	61.5
fold coverage	750	850	494	99
predicted ORFs	2,079	2,137	2,118	2,083
tRNAs	46	45	45	45
year isolated	1983	1991	1980	1978
location	South Australia	South Australia	Western Australia	Western Australia
host	*Lolium rigidum*	*Polypogon monspeliensis*	*Avena sativa*	*Phalaris paradoxa*
ALFP subgroup^1^	B	C	A	---

^1^ALFP subgroup designations were previously identified [11].

### Genome sequencing and assembly

For *R*. *toxicus* FH-79, a shotgun DNA library was constructed for the 454 Junior (Roche) according to the manufacturer’s directions and three sequencing runs were performed. In addition, a library FH-79 was also constructed for the PacBio RSII (Pacific Biosciences); three SMRT cells were sequenced for FH-79 at the Washington State University Genomics Lab. The 454 sequence data was assembled using Lasergene Ngen v12.0 (DNAStar) and PacBio reads using Pacific Bioscience’s Hierarchical Genome-Assembly Process (HGAP) [14]; consensus sequences from the two methods were compared using Ngen. For *R*. *toxicus* FH-232, only a PacBio library was constructed and 3 SMRT cells were sequenced also at the Washington State University Genomics Lab; assembly was performed using Pacific Bioscience’s Hierarchical Genome-Assembly Process (HGAP) [14]. The putative tunicamycin gene cluster, vancomycin resistance genes, and 16S rDNA from FH-79 and the CRISPR region from FH-232 were resequenced by primer walking on an Applied Biosystems 3130XL (Thermo Fisher Scientific) to validate genome assembly.

The genome sequences presented here have been deposited in GenBank under the following accession numbers: *R*. *toxicus* FH-79 BioProject PRJNA312185 and BioSample SAMN04495682; *R*. *toxicus* FH-232 BioProject PRJNA312185 and BioSample SAMN06040670.

### Genome annotation and analysis

Initial automated genome annotation was obtained using the Prokaryotic Genome Annotation Pipeline (PGAP) at National Center for Biotechnology Information (NCBI) [15]. Custom gene models were constructed as necessary by aligning the selected input sequences using muscle (http://www.drive5.com/muscle/) [16], followed by invocation of hmmbuild from the HMMer version 3.1.b2 package (http://hmmer.org/). The hmmscan tool from the HMMer suite was used for database scans. Predicted chromosomal origin of replication was identified using Ori-finder (http://tubic.tju.edu.cn/Ori-Finder/) [17]. Standard protein family and domain models were obtained from TBLASTN (https://blast.ncbi.nlm.nih.gov/Blast), Pfam (http://pfam.xfam.org/), TIGRFam (http://www.jcvi.org/cgi-bin/tigrfams/index.cgi) and TnpPred [18]. Alien_Hunter and antiSMASH were used to identify regions with anomalous nucleotide composition and putative biosynthetic clusters, respectively [19, 20]; identified regions were manually annotated with special attention paid to transposases and known virulence factors in other Actinobacteria. Whole genome alignments were performed with Mauve [21]. CRISPR analysis was performed using CRISPRFinder [22].

### Phylogenetic trees

For the Actinobacteria phylogenetic tree, sequences for *gyrB*, *secA1* and 16S rDNA genes were obtained for 15 representative species of Actinobacteria. Sequences were concatenated and aligned using three iterations of tree searching and realignment with the Clustal Omega algorithm in Megalign Pro (Lasergene). MEGA6 [23] was then used to conduct model determination and maximum likelihood tree searches (default settings) with 100 iterations of bootstrapping analyses. A minimum bootstrap value of 50 was used as a cut-off level of support to determine valid branches. *Rubrobacter radiotolerans* was set as the outgroup.

For the protease tree, amino acid sequences of serine proteases putatively secreted from *R*. *toxicus* FH-79 and *Clavibacter michiganensis* subsp. *michiganensis* NCPPB382 were aligned with MSAProbs [24, 25]. Aligned sequences were used to generate maximum-likelihood trees based on the Jones-Taylor-Thornton (JTT) model of MEGA 7.0 with bootstrapping repetitions of 1,000 [26, 27].

### GC content plot and statistics

Percentage GC content was plotted using GC content calculator (www.biologicscorp.com/tools/GCContent) with a sliding window size of 2,000 bp. Statistical significance of GC content differences was calculated by repeated random sampling of 1000 13.4 kb regions of the *R*. *toxicus* FH-79 genome excluding rDNA and the TGC itself.

## Results

### Whole-genome sequencing, assembly, and annotation

Sequence data resolved each genome into a single circular chromosome of 2,343,780 and 2,394,755 bp for *R*. *toxicus* FH-79 and FH-232, respectively; no plasmids or other extra-chromosomal sequences were found for either strain. The PacBio SMRT sequencing technology was especially important for evenly closing these high-GC genomes [28]. Table 1 compares these two genomes to the previously available *R*. *toxicus* FH-145 (NZ_CP010848) and WAC3373 [10]. All four *R*. *toxicus* strains have an average GC content of approximately 61%. Annotation using NCBI’s Prokaryotic Genome Annotation Pipeline yielded 2,078 open reading frames (ORFs) for *R*. *toxicus* FH-79 and 2,137 ORFs for FH-232 (Table 1). This PGAP annotation also contained a large number of genes with the \pseudo keyword due to variations in the placement of the stop codon. Manual comparison with carefully annotated genomes suggest that the observed variations in gene length are typical in Actinobacteria; therefore, the \pseudo keyword was removed.

**Fig 1:**
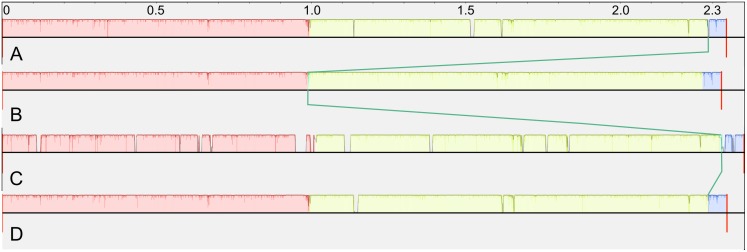
Collinearity of four complete *R*. *toxicus* genomes. A Mauve alignment shows two large locally collinear blocks separated by short transpositions. Green line connects short transposed region. A) *R*. *toxicus* FH-79; B) *R*. *toxicus* FH-232; C) *R*. *toxicus* FH-145 (NZ_CP010848.1); D) *R*. *toxicus* WAC3373 (NZ_CP013292.1).

The two sequenced genomes presented here were aligned with the two available *R*. *toxicus* genomes using Mauve [21] after rotating and/or reverse complementing sequences to place *dnaA* as the first gene on the positive strand. As shown in Fig 1, the four genomes are essentially syntenic. The pink, yellow, and blue regions represent three locally collinear blocks (LCBs). The distinction between the pink and blue regions is an artifact arising from circular genomes being treated as linear by the Mauve algorithm. Therefore, there are only two physically distinct LCBs separated by short transpositions; location of transposition region is marked by a green line in Fig 1. *R*. *toxicus* FH-232 has 12 insertions not present in the other genomes; this accounts for its larger genome size (Fig 1C).

Predicted and annotated open reading frames spanned the typical range of necessary biological functions, metabolism, cell wall biosynthesis, defense, etc. Importantly, no ORFs annotated as phage genes were present, indicating no prophages are incorporated into the bacterial genome and that samples were free from contaminating phage. *R*. *toxicus* FH-79 and FH-232 both have two 16S rDNA sequences and have 46 or 45 tRNAs, respectively (Table 1). Because of the extensive similarity among the four sequenced *R*. *toxicus* strains, further analysis is only presented for *R*. *toxicus* FH-79 except for rare cases where significant differences exist.

### *R*. *toxicus* groups with the *Microbacteriaceae*

**Fig 2:**
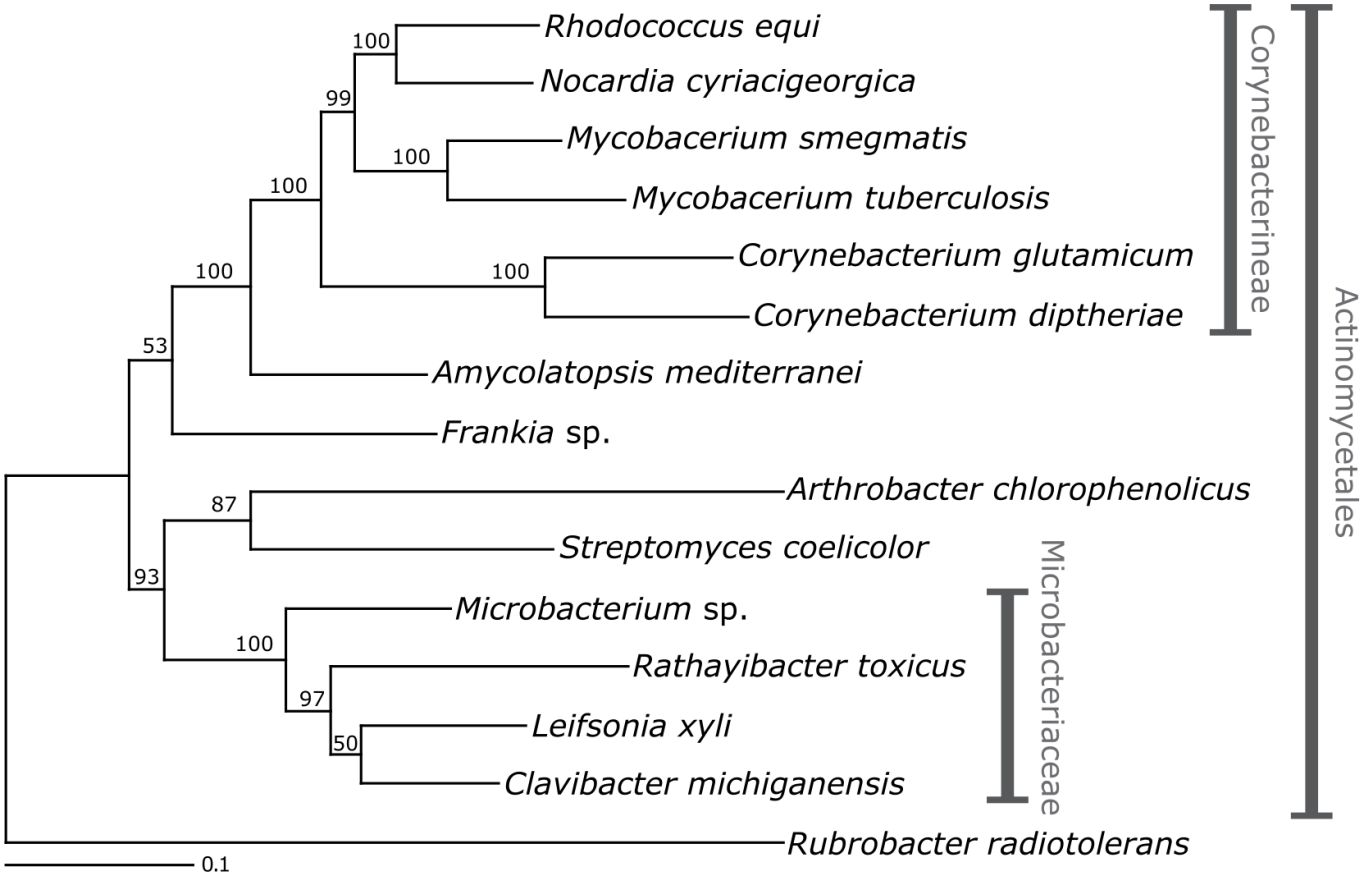
*R*. *toxicus* groups with the *Microbacteriaceae*. Maximum likelihood bootstrap phylogram of representative Actinobacteria showing strong support for placement of *R*. *toxicus* FH-79 in the *Microbacteriaceae*. Phylogeny based on concatenated 16S, *gyrB*, and *secA1* sequences.

A phylogenetic analysis based on three conserved genes clearly demonstrates that *R*. *toxicus* is a member of the *Microbacteriaceae*, most closely related to *Leifsonia xyli* and *Clavibacter michiganensis* (Fig 2). These three genes (*gyrB*, *secA1*, and 16S rDNA) are frequently used for resolving subfamilial relationships in Actinobacteria due to appropriate levels of within subfamily variation [29, 30]. Although *L*. *xyli* and *C*. *michiganensis* have slightly larger genomes than *R*. *toxicus* (2.6 Mb and 3.3 Mb, respectively, vs. 2.3 Mb), all three species have GC-rich genomes and all are plant-associated [31, 32].

### Tunicamycin gene cluster

**Fig 3:**
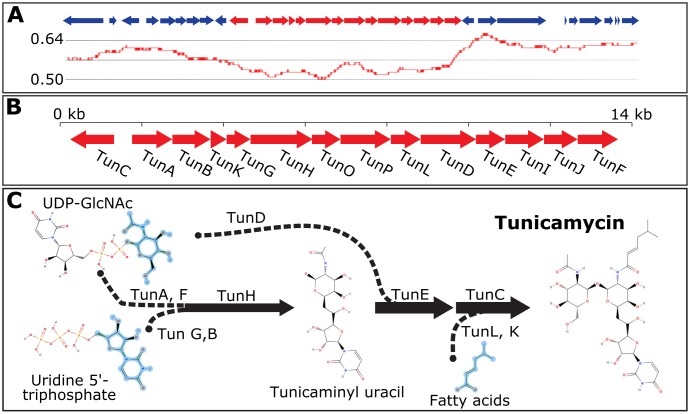
Structure of the tunicamycin gene cluster (TGC) from *R*. *toxicus* and overview of tunicamycin biosynthetic pathway. A) GC-content analysis of a 28-kb region surrounding the TGC. B) *R*. *toxicus* FH-79 TGC contains 12 genes with high homology to *tun* genes from *S*. *chartreusis* (*tunA-L*) and two additional genes (*tunO* and *P*) in two divergently transcribed operons. C) Hypothesized tunicamycin biosynthetic pathway. Incorporated fragments are highlighted in light blue. Adapted from [33, 34].

A putative 13.4 kb tunicamycin gene cluster (TGC) was identified based on homology to proteins encoded by the TGC from *Streptomyces chartreusis* NRRL 3882 [33]. As shown in Fig 3A, the *R*. *toxicus* TGC has a GC content markedly lower than the genome as a whole (52% vs. 61%). Repeated random sampling of the genome demonstrated that only 0.2% of comparably sized genome segments have a GC-content that is lower than the TGC (p-value < 0.002).

Although the *S*. *chartreusis* TGC appears to be a single polycystronic operon consisting of either 12 (*tunA-tunL*) [34] or 14 (*tunA-tunN*) [33] genes, the *R*. *toxicus* TGC contains two operons, one monocystronic (*tunC*) and one polycystronic (*tunA-tunF*; Fig 3B). *R*. *toxicus* also lacks the *tunM* methyltransferase and *tunN* NUDIX hydrolase; however, these genes are not essential for tunicamycin biosynthesis [34]. The TGC in *R*. *toxicus* does contain two novel ORFs: *tunO*, a hypothetical gene unique to *R*. *toxicus*, and *tunP*, a polyketide synthase with a beta-ketoacyl synthase domain. All the predicted TGC genes are present in the same order and orientation in all four sequenced strains. *R*. *toxicus* FH-145 and WAC3373 are identical at the nucleotide level to the FH-79 TGC except for the addition or deletion of 2 or 3 Gs in a highly repetitive, G-rich intergenic region upstream of *tunC*. The FH-232 TGC is more than 99% identical to the other TGC regions. FH-79 has been previously shown to produce tunicamycin [7]; FH-232 and FH-145 also produce toxin. While tunicamycin production by WAC3373 has not been reported, biosynthesis is likely given the highly conserved TGC. The hypothesized tunicamycin biosynthetic pathway is shown in Fig 3C [33, 34].

### Additional secondary metabolites

To identify regions with anomalous nucleotide composition that may interfere with statistically based gene calling algorithms, Alien_Hunter [19] was used to query the *R*. *toxicus* FH-79 genome. Such regions are also of interest because they may arise from horizontal gene transfer events and are more likely to contain biosynthetic genes for secondary metabolites or virulence factors. Forty-two regions, including the TGC described above, were identified and are listed in S1 Table. To further aid in the identification of secondary metabolite biosynthetic clusters, antiSMASH was also used to query the *R*. *toxicus* FH-79 genome [20]. As shown in S2 Table, 21 of the 42 regions identified with Alien_Hunter were also identified within 14 antiSMASH regions. Regions vary from 5.2–28.7 kb and are predicted to encode a wide variety of functions: bacteriocins (lantibiotic), type III polyketide synthase (PKS) proteins, non-ribosomal peptide synthetase (NRPS) proteins, multidrug efflux permeases, serine proteases, exopolysaccharide-related proteins, Type VII secretion system (T7SS) proteins, and numerous YD/RHS-like repeat-associated proteins.

**Fig 4:**
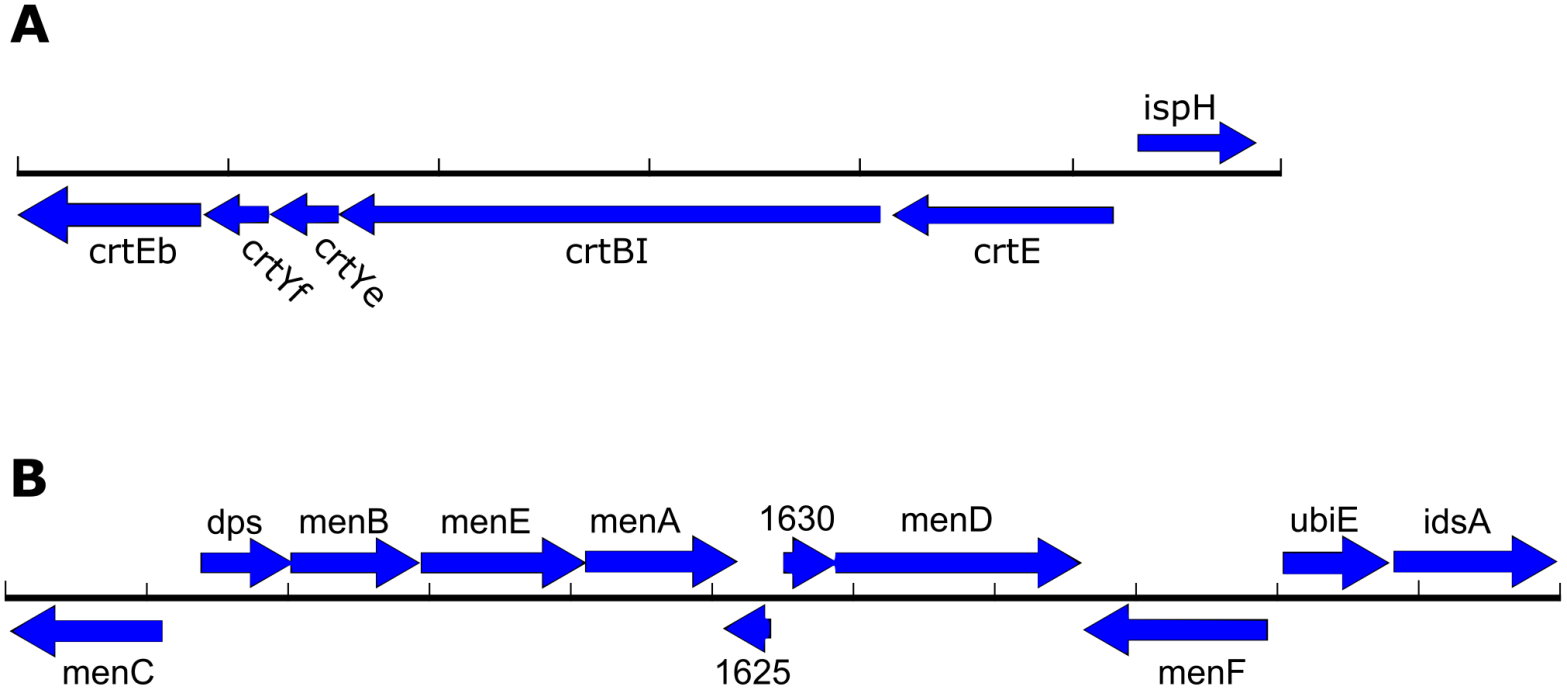
Pigment and menaquinone biosynthetic clusters. Gene clusters from *R*. *toxicus* FH-79 appearing to encode a carotenoid pigment (A) and menaquinone MK-10 (B). Scale bar ticks correspond to 1 kb.

Historically, *R*. *toxicus* has been defined based on several different biochemical characteristics. In addition to the production of tunicamycin as described above, these include yellow colony color, exopolysaccharide “slime” production, MK-10 as the predominant isoprenoid quinone, and a non-mevalonate pathway for isoprenoid biosynthesis [35, 36]. Although the exact biochemical nature of the yellow pigment has not been determined, the only candidate carotenoid biosynthetic cluster in the genome is shown in Fig 4A. It consists of six predicted genes: *crtEb* (AYW78_09695, UbiA-like prenyltransferase); *crtYf* (AYW78_09700, lycopene cyclase); *crtYe* (AYW78_09705, lycopene cyclase); *crtBI* (AYW78_09710, bifunctional phytoene synthase/oxidoreductase); *crtE* (AYW78_09715, geranylgeranyl diphosphate synthase); and *ispH* (AYW78_09720, isopentyl-diphosphate delta-isomerase, type I). The only predicted exopolysaccharide biosynthetic cluster in the *R*. *toxicus* genome is present on antiSMASH cluster AS-8 (S2 Table and S1A Fig). This cluster was identified based on similarity to proteins in the *wcm*, *wcn*, *wco*, and *wcq* exopolysaccharide biosynthetic clusters in *Clavibacter michiganensis* subsp. *nebraskensis* NCPPB 2581 (NC_020891.1). The carotenoid pigment and the secreted exopolysaccharide may account for the yellow slime observed during plant infection.

The menaquinone profile, along with 16S rDNA sequence and cell wall amino acid composition, was used to justify moving the type strain from *Clavibacter* to *Rathayibacter* [36]. The predominant menaquinone identified by Sasaki et al., MK-10, is also the expected product of a gene cluster from antiSMASH cluster AS-5 (Fig 4B). This cluster contains genes with similarity to *menB-menF* and *ubiE*, the core menaquinone biosynthetic genes first identified in *E*. *coli* [37], as well as several additional genes. The ORF labeled *idsA* is predicted to encode a geranylgeranyl pyrophosphate synthase that may be involved in both menaquinone and carotenoid production [38].

Most organisms use one of two different pathways to synthesize the important isoprenoid building blocks isopentenyl pyrophosphate and its isomer dimethylallyl pyrophosphate, either the classical mevalonic acid (MVA) pathway or the non-mevalonate/methylerythritol phosphate (MEP) pathway [39]. Although Gram-negative bacteria only use the MEP pathway, several Gram-positive organisms, including many in the *Microbacteriaceae* family, use the MVA pathway [35, 39]. Studies using the isoprenoid biosynthetic inhibitor fosmidomycin are consistent with use of the MEP pathway by several *Rathayibacter* species [35]. The *R*. *toxicus* genome contains ORFs similar to the core MEP pathway proteins from *E*. *coli*: DXS 1-deoxy-D-xylulose 5- phosphate synthase, AYW78_05260; DXR/IspC 1-deoxy-D-xylulose 5-phosphate reductoisomerase, AYW78_03715; IspE 4-diphosphocytidyl-2-C-methylerythritol kinase, AYW78_07950; and a bifunctional IspD/IspF 4-diphosphocytidyl-2-C-methylerythritol synthetase and 2-C-methylerythritol 2,4-cyclodiphosphate synthase, AYW78_08320. The MVA pathway appears to be absent from *R*. *toxicus*.

antiSMASH cluster AS-18 is predicted to encode a lantibiotic or class I bacteriocin, a heavily modified, ribosomally synthesized anti-microbial peptide [40]. The predicted prepropeptide is encoded by the gene with locus tag AYW78_09457 and is serine and alanine rich. Neighboring ORFs AYW78_09425 and AYW78_09430 encode proteins containing lantibiotic dehydratase domains while AYW78_09455 encodes a putative peptide cyclodehydratase (S1B Fig). AYW78_09440 and AYW78_09445 encode FMN-dependent oxidases that may act on the cyclized thioesters. The only *R*. *toxicus* gene that exhibits any significant similarity to the LanP-type peptidases involved in cleaving lantibiotic leader peptides is not part of this cluster (AYW78_08500).

Although not identified by either Alien_Hunter or antiSMASH, it is notable that the *R*. *toxicus* genome encodes three predicted vancomycin resistance proteins: VanH pyruvate dehydrogenase, AYW78_09940; VanA D-lactate dehydrogenase, AYW78_09945; and VanX D-ala-D-ala peptidase, AYW78_09950. *R*. *toxicus* FH-79 is resistant to vancomycin experimentally.

### CRISPR arrays

**Fig 5:**
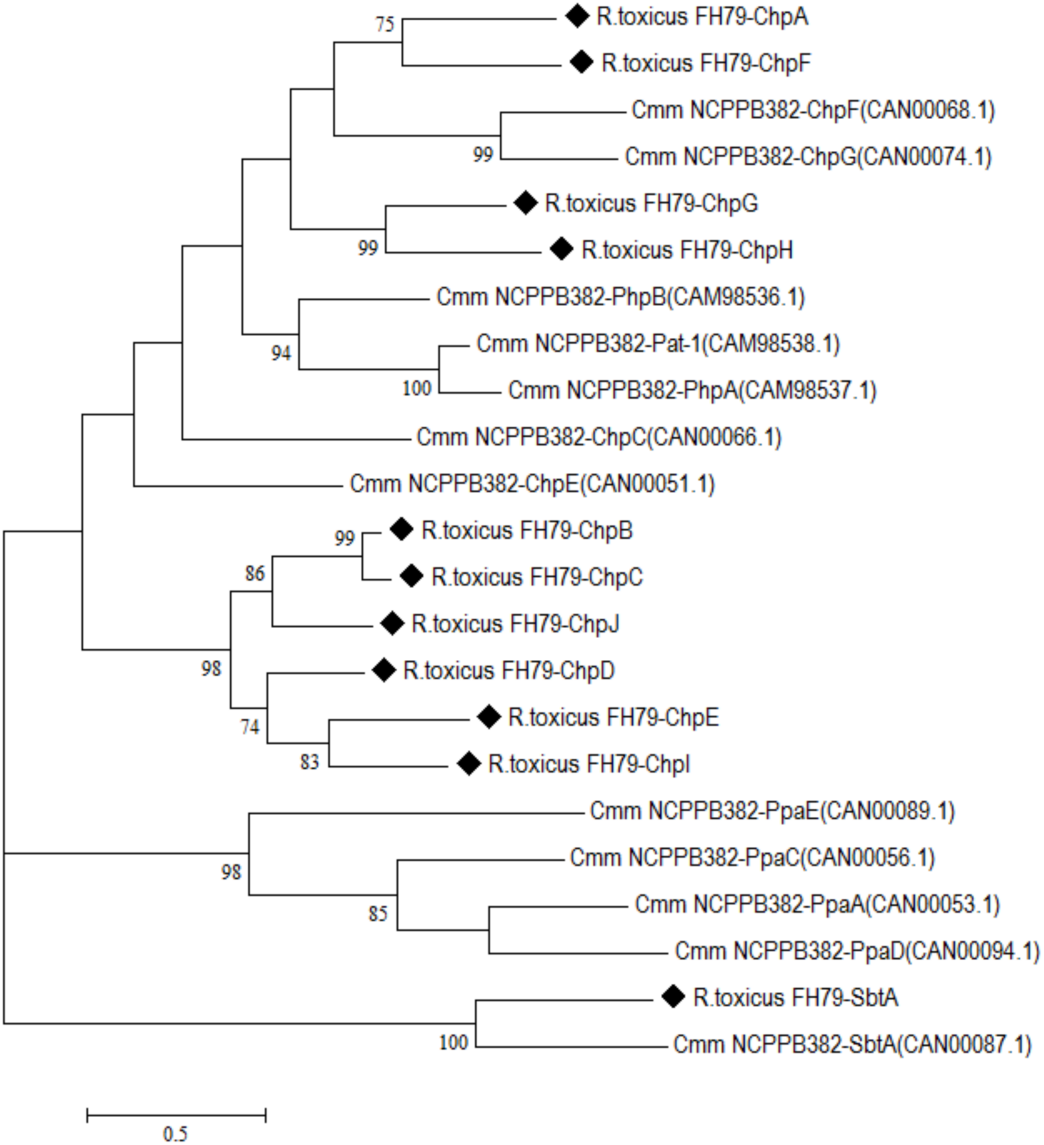
Maximum likelihood phylogenetic tree of putatively secreted serine proteases for *R*. *toxicus* FH-79 and *C*. *michiganensis* subsp. *michiganensis* NCPPB382. Percentage of trees in which the associated taxa clustered together is shown next to the branches; values less than 70 have been omitted. *R*. *toxicus* FH-79 is designated with black diamonds; gene name and accession numbers are displayed in parentheses.

*R*. *toxicus* possesses a complete Type I-E CRISPR-Cas system (*E*.*coli-*type) [41] with eight *cas* genes and an adjacent approximately 8.9 kb CRISPR spacer array (Fig 5A). The four different sequenced strains have slightly different numbers of non-repetitive spacer sequences and conserved direct repeats. *R*. *toxicus* FH-79 and FH-145 both have 145 non-repetitive spacer sequences and 146 conserved direct repeats while WAC3373 has 144 and 145 and FH-232 has 139 and 140, respectively. Non-repetitive spacer sequences revealed no identity to known plasmid or phage sequences.

### Predicted pathogenicity-related genes

Relative to the related phytopathogen *Clavibacter michiganensis* subsp. *michiganensis*, *R*. *toxicus* possesses a limited arsenal of plant-associated cell-wall hydrolyzing enzymes, consisting of only a single polygalacturonase (AYW78_01285) and pectate lyase (AYW78_01485). This is consistent with the life strategy of *R*. *toxicus*, which apparently cannot infect plant leaves or stems but most acquire nutrients in seed galls initiated by nematode infestation. However, *R*. *toxicus* does possess numerous secreted serine proteases that share sequence homology to the pathogenicity-associated protein Pat-1 of *C*. *michiganensis* subsp. *michiganensis*. A total of 11 secreted serine proteases were identified with an additional conserved pseudogene; all contain predicted signal peptides suggesting extracellular localization as described in *C*. *michiganensis* subsp. *michiganensis*. The corresponding genes were designated *chpA-K* (chromosomal homology to *pat*-1) and *sbtA* (subtilisin-like serine protease). In contrast to *C*. *michiganensis* subsp. *michiganensis*, the secreted serine proteases are dispersed throughout the chromosome, but several of the proteases are located in close proximity including: (i) *chpG*, *chpH*, *chpK* (pseudogene) and (ii) *chpB*, *chpC*.

**Fig 6:**
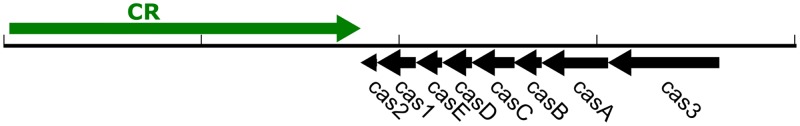
*R*. *toxicus* CRISPR locus. Coding regions are depicted with black arrows and the non-coding CRISPR is in green. Scale bar ticks correspond to 5 kb.

Phylogenetically, the serine proteases of *R*. *toxicus* and *C*. *michiganensis* subsp. *michiganensis* appear distinct with the majority of *R*. *toxicus* proteases (ChpB-E, ChpI-J) forming a subgroup (Fig 6). No *R*. *toxicus* serine proteases clustered with the *C*. *michiganensis* subsp. *michiganensis* Ppa family or plasmid-associated (PhpA-B) serine proteases. The subtilisin-like serine proteases of *R*. *toxicus* and *C*. *michiganensis* subsp. *michiganensis* were the only secreted proteases to cluster across species (Fig 6).

## Discussion

The key feature of *R*. *toxicus* is its ability to exploit a protected environmental niche, the developing grass seed, and produce tunicamycin, a potent toxin for grazing livestock. Prior to the work presented here, very little was known about the biosynthesis of tunicamycin by *R*. *toxicus*. Until the publication of the phage NCPPB 3778 sequence [9], it was hypothesized that tunicamycin production could reside in the phage rather than on the bacterial chromosome. However, no ORFs with similarity to known tunicamycin biosynthetic genes were found in the phage genome [9]. The discovery of a tunicamycin gene cluster (TGC) in *R*. *toxicus* (Fig 3) with similarity to the previously characterized cluster from *S*. *chartreusis* is an important first step in understanding toxin production in this bacterium. Both the lower GC content of the TGC and its similarity to *Streptomyces* indicate that *R*. *toxicus* probably acquired the ability to synthesize tunicamycin via a horizontal gene transfer event; however, the TGC does not contain identifiable transposases, nor is it adjacent to a recognizable tRNA or flanked by inverted repeats as is typical for a mobile genetic element.

*R*. *toxicus* is regulated as a select agent because it is associated with the production of toxin that results in the death of foraging livestock. There are additional concerns about potential secondary effects that could manifest in humans consuming either contaminated plant material or the meat of ARGT affected animals. *R*. *toxicus* causes little in the way of disease symptoms on grasses, with the accumulation of exopolysaccharide “slime” as the primary sign of pathogen infection, and there is no indication that *R*. *toxicus* infections result in significantly reduced plant host fitness. The lack of phytopathogenesis-related genes in the *R*. *toxicus* genome further suggests that this bacterial species may not be a typical plant pathogen. Rather, *R*. *toxicus*, like other *Rathayibacter* species, has evolved a unique approach to reaching and exploiting a desirable niche, by utilizing gall forming nematodes as a convenient vector.

A possible biological function for toxin production is the elimination of nematodes from the seed gall, thus eliminating competition for resources. Tunicamycin production increases drastically when *R*. *toxicus* is inside the seedhead at a tipping-point between the nascent gall progressing to either nematode or bacterial dominated growth [42]. However, while all members of the *Rathayibacter* genus utilize gall forming nematodes as vectors not all members of the genus produce tunicamycin, although it is not yet known whether the TGC is present in all members of the genus. It should be noted that toxin production comes at a significant fitness cost to *R*. *toxicus*, as toxin producing bacteria reproduce at significantly slower rates than non-toxin producers [6]. Alternatively, toxin production for *R*. *toxicus* may provide an advantage against competing microbial populations, both fungal and bacterial, at one or more points in the life cycle from soil to seed head. Microbial competition could also explain the repertoire and diversity of biosynthetic pathways encoding non-ribosomal peptide synthetase (NRPS) proteins, polyketide synthase (PKS) proteins, thiazole/oxazole-modified microcins, lantibiotics, and numerous efflux proteins present in the *R*. *toxicus* genome. Regardless, for the select agent *R*. *toxicus*, there would seem to be some selection pressure(s) acting to keep the TGC and associated machinery present and active in the bacterial genome.

It is not known how any tunicamycin producer protects itself from the toxin. It has been hypothesized that *tunI* and *tunJ*, which are both similar to ABC transporters, export tunicamycin outside the cell immediately after synthesis [33, 34]. It is possible to express the *S*. *chartreusis* TGC in other *Streptomyces* species and thereby confer both tunicamycin production and resistance, implying that at least in the case of *S*. *chartreusis*, any export or detoxification mechanisms reside within the TGC itself [33, 34].

The *R*. *toxicus* strains sequenced here complement the two previously available complete genome sequences. A previous analysis of *R*. *toxicus* strains found three major genotypic groups based on amplified fragment length polymorphisms (AFLP) and restriction digestion patterns using pulsed-field gel electrophoresis (PFGE). As indicated in Table 1, the previously sequenced *R*. *toxicus* FH-145 falls in subgroup A while FH-79 is in subgroup B and FH-232 is the sole member of subgroup C. Many of the same *R*. *toxicus* strains, as well as some more recently collected, were also analyzed by multi-locus sequence typing (MLST) and inter-simple sequence repeats (ISSR) [10]. This analysis found three main populations, RT-I, RT-II, and RT-III, with strain FH-232/FH100 again forming an outgroup. *R*. *toxicus* FH-79 and FH-145 were not included in the MLST analysis. However, by *in silico* PCR, they both belong to RT-III. The four subgroup A strains also analyzed by MLST all fall into RT-III while the three subgroup B strains examined are in RT-II. This makes *R*. *toxicus* FH-79, which is the type strain for the species [43], somewhat unusual as it falls into subgroup B and RT-III.

It is most common for bacterial chromosomes to be circular in topology. However, a number of both Gram-positive and Gram-negative bacteria have linear chromosomes and/or plasmids; they are especially common in the *Actinomycetales* [44]. The *R*. *toxicus* chromosome was hypothesized to be linear based on its failure to enter a pulsed-field gel either before or after nuclease S1 treatment [11]. Whether or not large circular DNA migrates during PFGE depends on the exact electrophoretic conditions [45]; insufficient experimental detail is provided to assess the conclusions of Agarkova *et al*. [11]. The genome presented here is most consistent with a circular topology. Virtual *Pac*I digests of a circular genome generate the number and size of bands observed experimentally more closely than a linear genome [11]. Additional bands are predicted but would not be expected to be visible on a pulsed-field gel due to their small size.

Linear chromosomes have large terminal inverted repeats on the ends; these sequences can be up to 1 Mb each [46]. Unless care is taken during genome assembly, these terminal repeats can be mis-assembled on top of each other and give the appearance of a circular genome. Terminal repeats have been observed to be under-represented in PacBio raw reads, perhaps because of the bias toward long DNA fragments during library construction [46]. Therefore one clue that a genome is linear can be the presence of contigs made up of short (Illumina or 454) reads that do not map to PacBio consensus sequence; no such contigs were found in the 454 sequence from *R*. *toxicus* FH-79. If terminal repeats are incorporated into the PacBio library and therefore appear once in a circular consensus sequence, those regions would be overrepresented in short read libraries. However, no such regions of higher coverage were observed.

Prior estimates of genome size [11] match the sequence obtained here quite well (2.2–2.3 Mb predicted vs. 2.3–2.4 Mb observed); if two large terminal repeats were missing from the genomes reported here, the sequences reported here would be expected to be significantly smaller than previous size predictions. In general, the larger *Actinomycetales* genomes tend to be linear and the smaller ones circular, although there are exceptions [44]. *R*. *toxicus*, at 2.3–2.4 Mb, is definitely on the smaller end of genome size. All of these factors taken together tend to support the presence of a circular chromosome in *R*. *toxicus*.

*R*. *toxicus* is most closely related to the systemic xylem-dwelling Gram-positive phytopathogenic bacteria *Clavibacter michiganensis* and *Leifsonia xyli*. While *L*. *xyli* subsp. *xyli* is a fastidious xylem-limited bacterium of sugarcane, *C*. *michiganensis* subsp. *michiganensis* is an opportunistic pathogen of tomato and colonizes both vascular and non-vascular tissue [31, 32]. Regardless of differences in host and systemic lifestyles, *C*. *michiganensis* subsp. *michiganensis* and *L*. *xyli* subsp. *xyli* possess numerous canonical plant-associated cell wall-degrading enzymes (PCWDEs) [31, 32, 47]. *C*. *michiganensis* subsp. *michiganensis* utilizes a variety of PCWDEs including hemicellulases, xylanases, cellulases, polygalacturonases, pectate lyases, and endoglucanases [31]. However, *R*. *toxicus* lacks many PCWDEs, possessing only a single copy each of pectate lyase and polygalacturonase. The relatively small arsenal of plant-associated enzymes is surprising for a plant pathogen, but could demonstrate its closer association and reliance on a nematode vector for plant colonization.

Despite the small arsenal of PCWDEs, *R*. *toxicus* possesses numerous serine proteases with homology to the pathogenicity-associated protein Pat-1 of *C*. *michiganensis* subsp. *michiganensis*. *C*. *michiganensis* subsp. *michiganensis* harbors serine proteases on a putative 129 kb pathogenicity island and extra-chromosomal plasmids, which are necessary for effective disease development in tomato [31, 48]. However, the serine proteases from *R*. *toxicus* are dispersed throughout the chromosome and appear distinct from the *C*. *michiganensis* subsp. *michiganensis* disease-associated serine proteases. The putatively secreted *R*. *toxicus* serine proteases could possess alternative functions associated with nematode colonization, as opposed to plant colonization or disease development, since cuticle penetrating serine proteases are highly represented in nematode pathogenic bacteria and fungi [49, 50]. It is interesting to note that Bird et al. (1984 & 1985) document the destruction of the nematode epidermis and cortical structures shortly after *Rathayibacter* attachment [51, 52]. The relative lack of PCWDEs and differing serine proteases suggest that *R*. *toxicus* is not a typical vectored phytopathogenic bacterium.

In summary, analysis of the complete genome of *R*. *toxicus* has identified a likely genetic pathway (TGC) for the production of tunicamycin, based on homology to other tunicamycin biosynthetic clusters. This represents a critical first step towards understanding the control of the key pathway that makes this Select Agent pathogen such a significant threat to agriculture and food safety. Sequencing the genomes of other members of the *Rathayibacter* genus, both toxin producers and non-toxin producers, would provide corroborative evidence implicating the TGC in tunicamycin production as well as providing some evolutionary context for the introduction of the TGC as a likely mobile element. The current genomic context, however, suggests that the TGC is no longer mobile in any of the sequenced *R*. *toxicus* strains. Ultimately, the connection between the TGC and toxin production must be assessed by expression studies, gene knockouts, and functional restoration experiments.

## Supporting information

S1 Fig Exopolysaccharide and lantibiotic biosynthetic clusters. Gene clusters from *R*. *toxicus* FH-79 appearing to encode exopolysaccharide biosynthesis (A) and a bacteriocin or lantibiotic (B). Scale bar major ticks correspond to 5 kb, minor tics 1 kb.
(PDF)

S1 Table AlienHunter regions. Genomic regions of interest putatively acquired through horizontal gene transfer events for *R*. *toxicus* FH-79 and identified with the data-mining software AlienHunter.
(PDF)

S2 Table Anti-smash regions. Genomic regions putatively involved in antibiotic and secondary metabolite production for *R*. *toxicus* FH-79 and identified with the data-mining software antiSMASH v. 3.0.
(PDF)
