# Supplementary figures and images for: Whole genome sequence of two Rathayibacter toxicus strains reveals a tunicamycin biosynthetic cluster similar to Streptomyces chartreusis
Source: PLoS One. 2017 Aug 10;12(8):e0183005. doi: 10.1371/journal.pone.0183005 (PMC5552033; doi:10.1371/journal.pone.0183005)

**A**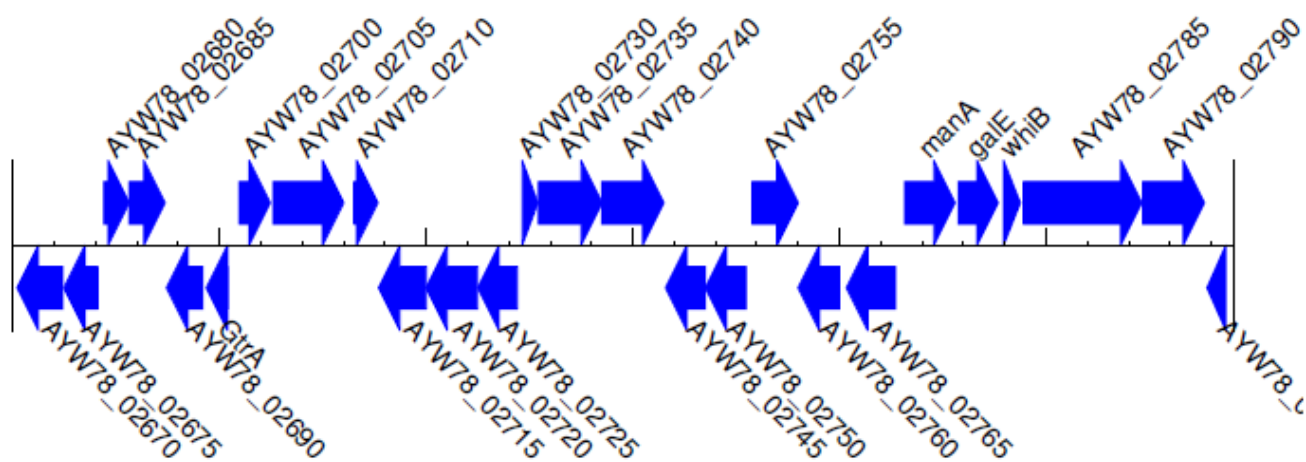**B**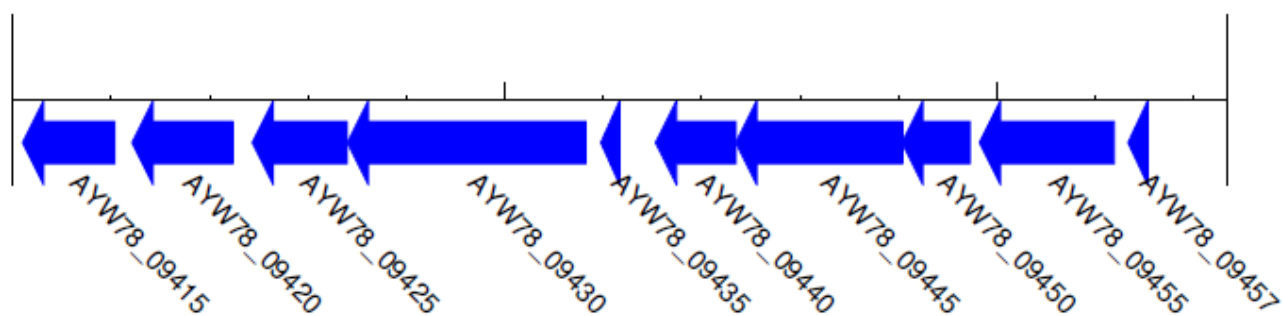

Supplement: S1 Fig — Gene clusters from R. toxicus FH-79 appearing to encode exopolysaccharide biosynthesis (A) and a bacteriocin or lantibiotic (B). Scale bar major ticks correspond to 5 kb, minor tics 1 kb. (PDF) [file pone.0183005.s001.pdf]
