# Supplementary material for: Whole genome sequence of two Rathayibacter toxicus strains reveals a tunicamycin biosynthetic cluster similar to Streptomyces chartreusis
Source: PLoS One. 2017 Aug 10;12(8):e0183005. doi: 10.1371/journal.pone.0183005 (PMC5552033; doi:10.1371/journal.pone.0183005)
Supplement: S1 Table — Genomic regions of interest putatively acquired through horizontal gene transfer events for R. toxicus FH-79 and identified with the data-mining software AlienHunter. (PDF) [file pone.0183005.s002.pdf]

Supplemental Table 1: Genomic regions of interest putatively acquired through horizontal gene transfer events for *Rathayibacter toxicus* FH-79 and identified with the data-mining software AlienHunter (Vernikos and Parkhill 2006).

Regions of interest	% GC	Size (kb)	Coordinates	Putative gene(s) of interest	Putative role
AH-1	61	14.4	54051- 68462	Glycosyltransferases; phosphatases; multidrug efflux proteins	Secondary metabolite
AH-2	58	11	85535- 96547	Metalloprotease; multidrug efflux proteins	Secondary metabolite
AH-3	53	10.8	142893- 153730	Multidrug efflux proteins; phosphates; epimerase	Secondary metabolite
AH-4	54	13.8	217546- 231327	YD/RHS-like repeat associated proteins	Secondary metabolite
AH-5	56	4.7	327699- 332435	Transposase	UNK
AH-6	61	10.8	348209- 359023	Pectate lyase	Virulence
AH-7	57	9.4	435885- 445293	Transglycosylase	UNK
AH-8	59	8.5	498510- 506994	Type VII secretion-like protein; Toll-interleukin 1-resistance (TIR) domain-containing protein; plasmid stabilization proteins	Secondary metabolite; toxin-antitoxin system
AH-9	61	9	507607- 516588	Multidrug efflux proteins; Nicotinamidase-related amidase	UNK
AH-10	57	18.7	630151- 648865	YD/RHS-like repeat associated proteins; Thiazole/oxazole-modified microcins (TOMMs)	Polyketide/ secondary metabolite
AH-11	60	20.5	650601- 671060	Type VII secretion proteins; YD/RHS-like repeat associated proteins; chitinase-like protein	Secretion/ secondary metabolite
AH-12	72	11.9	736861- 748754	Non-ribosomal peptide synthetase; multidrug efflux proteins; peptidase	NRPS/ secondary metabolite
AH-13	59	8.5	754719- 763195	ABC transporters	Secondary metabolite
AH-14	49	7.1	848056- 855140	Transposase	UNK
AH-15	56	8.9	873535- 882442	ChpB; ChpC	Serine protease/virulence
AH-16	52	13.4	905229- 918630	Tunicamycin-like genes	Tunicamycin/Corynetoxin
AH-17	57	5.2	941897- 947133	ChpD	Serine protease/virulence
AH-18	54	18.1	979717- 997780	YD/RHS-like repeat associated proteins; bacterial EndoU nucleases	Secondary metabolite
AH-19	61	12.1	1086846- 1098917	Multidrug efflux proteins; lantibiotic cyclase; transposase	Secondary metabolite
AH-20	55	16.2	1252302- 1268522	Multidrug efflux proteins; acyl transferases	Secondary metabolite
AH-21	60	8.3	1349072- 1357411	Multidrug efflux proteins; patatin-like phospholipase	Secondary metabolite

AH-22	58	9.3	1374127- 1383439	Bacterial lysin (BacA-like); Multidrug efflux proteins	Secondary metabolite
AH-23	55	5.3	1416316- 1421650	ChpE	Serine protease/virulence
AH-24	59	13.3	1427419- 1440749	Cytochrome C oxidase	Cytochrome C oxidase gene cluster
AH-25	60	13.2	1501861- 1515067	Nucleotidyl transferase AbiEii toxin; transposase	toxin-antitoxin bacterial abortive infection system
AH-26	59	11.9	1608300-1620201	Type VII secretion proteins; DNA/RNA non-specific endonuclease	Secretion/ secondary metabolite
AH-27	64	18.2	1623809-1642017	LPS heptosyltransferase; glycosyl transferases	Cell wall/EPS biogenesis (similarity to *Leucobacter* sp. LPS)
AH-28	59	9.6	1645206- 1654758	YD-like repeat and RHS repeat-associated core domain-containing proteins; bacterial EndoU nuclease	Secondary metabolite
AH-29	67	11.5	1768456- 1779983	ChpF; glycosyl transferase	Serine protease/virulence
AH-30	54	6.3	1789681-1795938	putative AbiEii toxin	toxin-antitoxin bacterial abortive infection system
AH-31	57	10.3	1925902-1936175	ATP-grasp domain-containing proteins; phosphoenolpyruvate mutase	UNK
AH-32	56	9	1979294-1988294	Hypothetical (pillus-like protein)	UNK
AH-33	59	12.8	1998528- 2011337	Multidrug efflux proteins	Secondary metabolite
AH-34	58	8.8	2031732- 2040560	Serine proteases (ChpG, ChpK, ChpH); Endonuclease/Exonuclease/phosphatase family-like protein	Serine protease/virulence
AH-35	58	11.3	2044513-2055855	ATP-grasp domain-containing proteins; phosphoenolpyruvate mutase	Secondary metabolite
AH-36	62	7.8	2139354- 2147203	Subtilisin-like peptidase	UNK
AH-37	58	7.4	2155869-2163313	hypothetical protein repeat	UNK
AH-38	58	20	2208670-2228730	Type VII secretion proteins; bacteriocin efflux proteins; ChpJ; transposase	Secretion/ secondary metabolite
AH-39	53	12.3	2239582-2251891	Multidrug efflux proteins; glycosyl transferases	Secondary metabolite
AH-40	58	11.2	2276281-2287447	YD/RHS-like repeat associated proteins; bacterial EndoU nucleases	Secondary metabolite
AH-41	54	7.7	2308630-2316348	Multidrug efflux proteins	Secondary metabolite
AH-42	57	12.9	2330379-2343253	chromosome partioning proteins; membrane proteins	UNK
