# Supplementary material for: Whole genome sequence of two Rathayibacter toxicus strains reveals a tunicamycin biosynthetic cluster similar to Streptomyces chartreusis
Source: PLoS One. 2017 Aug 10;12(8):e0183005. doi: 10.1371/journal.pone.0183005 (PMC5552033; doi:10.1371/journal.pone.0183005)
Supplement: S2 Table — Genomic regions putatively involved in antibiotic and secondary metabolite production for R. toxicus FH-79 and identified with the data-mining software antiSMASH v. 3.0. (PDF) [file pone.0183005.s003.pdf]

Supplemental Table 2: Genomic regions putatively involved in antibiotic and secondary metabolite production for *Rathayibacter toxicus* FH-79 and identified with the data-mining software antiSMASH v. 3.0 (Weber et al. 2015).

Regions of interest	Putative gene cluster	% GC	Size (kb)	Coordinates	Putative gene(s) of interest	AlienHunter overlap
AS-1	-	61	14.4	54051- 68462	Glycosyltransferases; phosphatases; multidrug efflux proteins	AH-1
AS-2	A	58	11	85535- 96547	Metalloprotease; multidrug efflux proteins	AH-2
AS-2	B	63	13.3	106920- 120228	Multidrug efflux proteins; exodeoxyribonuclease III (similarity to Natamycin biosynthetic gene cluster)	-
AS-3	A	62	6.1	182853- 188920	Type III polyketide synthase	-
AS-3	B	64	11.2	200753- 211986	Amino acid permeases; glycosyltransferases	-
AS-3	C	54	13.8	217546- 231327	YD/RHS-like repeat associated proteins	AH-4
AS-4	-	64	9.4	318313- 327700	Peptide synthase; 3-oxoacyl-ACP synthase III	-
AS-5	-	65	12.2	372485- 384693	Menaquinone biosynthetic cluster	-
AS-6	A	61	5.7	411139- 416791	Amino acid permease; two-component system proteins (similarity to clavulanic acid biosynthetic gene cluster)	-
AS-6	B	57	9.4	435885- 445293	Transglycosylase	AH-7
AS-7	-	60	7.6	470870- 478475	exopolysaccharide biosynthesis- related genes	-
AS-8	-	63	28.7	582536- 611266	Wcq-like exopolysaccharide biosynthetic gene cluster	-
AS-9	-	63	7.4	696648- 704027	Aminotransferases; enoylreductase (polyketide synthesis)	-
AS-10	A	72	11.9	736861- 748754	Non-ribosomal peptide synthetase; multidrug efflux proteins; peptidase	AH-12
AS-10	B	59	8.5	754719- 763195	ABC transporters	AH-13
AS-11	-	61	19.2	790328- 809488	Glycosyltransferases; methyltransferase	-
AS-12	A	52	13.4	905229- 918630	Tunicamycin-like genes	AH-16
AS-12	B	62	14.4	924102- 938484	Non-ribosomal peptide synthetase; multidrug efflux proteins	-
AS-12	C	57	5.2	941897- 947133	ChpD	AH-17
AS-13	A	61	12.1	1086846- 1098917	Multidrug efflux proteins; lantibiotic cyclase; transposase	AH-19
AS-13	B	62	21.4	1102200- 1123610	beta-ketoacyl-ACP reductase; glycosyltransferases	-
AS-14	-	55	16.2	1252302- 1268522	Multidrug efflux proteins; acyl transferases	AH-20
AS-15	A	60	8.3	1349072- 1357411	Multidrug efflux proteins; patatin-like phospholipase	AH-21
AS-15	B	63	7.7	1359348- 1367067	Acyl-carrier proteins; PucR family transcriptional regulator	-

AS-15	C	58	9.3	1374127- 1383439	Bacterial lysin (BacA-like); Multidrug efflux proteins	AH-22
AS-16	A	59	11.9	1608300-1620201	Type VII secretion proteins; DNA/RNA non-specific endonuclease	AH-26
AS-16	B	64	18.2	1623809-1642017	LPS heptosyltransferase; glycosyltransferases	AH-27
AS-16	C	59	9.6	1645206- 1654758	YD-like repeat and RHS repeat-associated core domain-containing proteins; bacterial EndoU nuclease	AH-28
AS-17	A	67	11.5	1768456- 1779983	ChpF; glycosyltransferase	AH-29
AS-17	B	54	6.3	1789681-1795938	putative AbiEii toxin	AH-30
AS-17	C	63	16.3	1796651- 1812920	polyketide cyclase	-
AS-18	A	59	9.2	2031332- 2040560	Serine proteases (ChpG, ChpK, ChpH); Endonuclease/Exonuclease/phosphatase family-like protein	AH-34
AS-18	B	58	11.3	2044513-2055855	ATP-grasp domain-containing proteins; phosphoenolpyruvate mutase (similarity to Rhizocticin biosynthetic gene cluster)	AH-35
AS-18	C	64	12.9	2065102- 2077962	Lantibiotic: bacteriocin biosynthesis cluster	-
AS-18	D	62	7.6	2087211- 2094820	Antibiotic biosynthesis monooxygenase; multidrug efflux protein; CheY-like REC domain-containing protein	-
AS-19	-	53	12.3	2239582-2251891	Multidrug efflux proteins; glycosyltransferases	AH-39
AS-20	-	54	7.7	2308630-2316348	Multidrug efflux proteins	AH-41
